# Matrix Description of Non-Linear Properties of Materials or Structural Components—Idea and Application Examples

**DOI:** 10.3390/ma14195837

**Published:** 2021-10-06

**Authors:** Tomasz Janiak

**Affiliations:** Faculty of Civil and Environmental Engineering and Architecture, Bydgoszcz University of Science and Technology, 85-796 Bydgoszcz, Poland; tomasz.janiak@pbs.edu.pl

**Keywords:** constitutive model, elastoplastic materials, numerical simulations

## Abstract

Numerical methods are widely used in structural analysis problems. In the cases of the most complex and practical problems, they are often the only way to obtain solutions, as analytical methods prove ineffective. The motivation for this paper was the desire to extend the scope of numerical methods to cover the problems of creating constitutive models of structural materials. The aim of this research was to develop a matrix or numerical discrete constitutive model of materials. It presents the general assumptions of the developed method for modeling the physical properties of materials. The matrix model is only useful with an appropriate numerical algorithm. Such an algorithm was created and described in this paper. Based on its findings, computer software was developed to perform numerical simulations. Presented calculation examples confirmed the effectiveness of the developed method to create constitutive matrix models of various typical materials, such as steel, but also, e.g., hyper-elastic materials. It also presents the usefulness of constitutive matrix models for simulations of simple stress states and analyses of structural elements such as reinforced concrete. All presented examples involved the physical nonlinearity of the materials. It is proved that the developed matrix constitutive model of materials is efficient and quite versatile. In complex analyses of structures made of nonlinear materials, it can be used as an effective alternative to classical constitutive or analytical models based on elementary mathematical functions.

## 1. Introduction

Structural materials used in the construction industry feature a number of parameters that are important primarily in terms of material strength and structure mechanics, but also in terms of other parameters related to, for example, building physics. One of the basic parameters is the material stiffness that is necessary to determine displacements and forces in structural systems.

Basic construction materials (steel and concrete in particular) behave in a non-linear way through the whole operating range. This means that their stiffness changes along with the increase of strain and stress. In the process of designing steel structures, the amount of stress or strain is limited to a certain acceptable level. This level is related to material specification (e.g., a clear yield point) or it is a defined arbitrary limit. This approach means that steel structures almost always operate in constant stiffness range and it is justified to assume the constant material stiffness in static calculations. The situation is completely different in case of reinforced concrete structures. Here, cracks occur very quickly (at a relatively low level of internal forces) in the tensile zones of concrete cross-sections. As a result, tensile stresses in the cross-sections of bent reinforced concrete structural elements are mainly transferred with reinforcement bars, and compressive stresses are mainly transferred with concrete. When internal forces increase, concrete or steel becomes plasticized, i.e., it behaves in a non-linear way. Yet, most static analyses of reinforced concrete structures are carried out in a simplified way, assuming that the geometric characteristics of cross-sections correspond to an uncracked concrete cross-section (without any reinforcement). This primarily applies to engineering calculations. It leads to displacement values even several times lower compared to the values experienced in real life. In statically indeterminable systems, significant errors also appear in the results of internal forces.

The simulation of actual behavior of reinforced concrete requires non-linear calculations. Such analyses have been carried out for decades, but have also recently been the subject of many research studies. For example, Dudziak [1] presented the non-linear analysis of reinforced concrete structures for engineering applications and proposed a new constitutive hypoelastic–brittle model of concrete. Yapar et al. [2] carried out numerical simulations of the behavior of prefabricated pre-tensioned concrete beams in specific phases of operation, also after exceeding limit states (which resulted in damage). The calculation took into account the non-linear, degradation, elastic–plastic concrete model and the slip between bars and concrete. Lou et al. [3] performed numerical analyses of double-span post-tensioned reinforced concrete beams with a non-linear course of prestressing tendons. They took into account non-linear constitutive relations for concrete, tensioned reinforcing steel and un-tensioned reinforcing steel. The analyses took into account the effects of concrete creep and concrete shrinkage, and tendon relaxation. Material non-linear properties were taken into account in the analysis of composite elements where steel profiles interacted with a concrete cross-section. Chiorean and Buru [4] developed a method for non-linear analysis of beams consisting of a steel I-beam profile and a reinforced concrete slab (the connection between elements was non-continuous and used steel shear connectors. Liang and Fragomeni [5] in turn were engaged in the non-linear analysis of columns consisting of concrete-filled steel pipes. Richard et al. [6] also worked with reinforced concrete beams, but they focused on the modeling of corroded reinforcement. Their paper formulated and used a non-linear steel/concrete interface constitutive law. Non-linear behavior of steel and concrete was also taken into account. Non-linear analyses of constant curvature cantilever beams loaded with follower force at the end [7] and non-linear calculations were also performed in studies related to less typical structural components. Zhang et al. [8] analyzed displacements of geocell mattresses that are a kind of geosynthetic reinforcements of the ground, e.g., beneath an embankment. In this case, a geometrically non-linear beam resting on non-linear subgrade soil was taken as the calculation model for geocell mattress. Davids [9] in turn analyzed arch structures made of inflatable (pressurized) fabric circular profiles. Displacements and buckling of such arches were analyzed. The material starts to wrinkle under loads, which was modeled by adopting a non-linear moment–curvature relation. Patel et al. [10] have performed a non-linear analysis of micro-beams. They were structural elements of micro-electro-mechanical systems (MEMS) and nano-electro-mechanical systems (NEMS). A non-linear analysis of the behavior of beam-to-column connection zones in precast structures was also performed [11]. Various topics related to concrete structures were also discussed in the research papers [12,13,14,15,16].

Many researchers have been involved in the modeling of hyper-elastic materials. Rubber and rubber-filled compounds are best examples of such materials. Hyper-elastic materials can experience large elastic strains and behave non-linearly in the entire operating range. Therefore, correct modeling of constitutive relations is essential for the analysis of stresses and displacements that are produced under loads. Many review and comparison articles have been created on models of hyper-elastic constitutive materials (e.g., [17,18,19,20]). New or improved models are continually being developed. Liu and Hoo Fatt [21] developed a hyper-viscoelastic constitutive equation to simulate steady-state response of rubber-filled compounds subjected to cyclic loads. A number of works of a similar nature were created (e.g., [22,23]). A six-parameter model, distinguished by its complexity, was developed by Nguessong Nkenfack et al. [24]. Hyper-elastic models are also suitable for modeling soft body tissues. This case was discussed by, e.g., Zanelli et al. [25] with modeling the urethral tissue. They carried out complex analyses to determine optimal values and the number of relevant model parameters. The issues related to the analysis of various hyper-elastic materials were also discussed in the articles [26,27,28,29,30].

The discussed studies were mainly related to two issues: non-linear analyses of construction elements (mainly reinforced concrete) and non-linear models of hyper-elastic constitutive materials and their utilization. In some cases, the cyclic load of the system that usually generates a hysteretic response is taken under consideration ([6,10,11,21,30,31]). The studies feature a common theme that is creating models of constitutive materials or elements—using algebraic or differential mathematical relations describing the stress–strain or moment–curvature relation. Whether models in analyses are correct was most often verified by comparison with experiment results or other independent numerical calculations of the same issue. The desired situation was the high compatibility of the obtained results with the experiment, which could not always be achieved. Moving towards the increased compatibility of the constitutive model and actual material behavior during the experiment, Sussman and Bathe [32] proposed an alternative approach to describe the physical properties of the material. They presented the concept of constructing a constitutive relation in the form of a spline function, consisting of segments of a cubic spline. This enables us to achieve a very high consistency with the results of experimental research. The Sussman and Bathe concept was used in other authors’ studies [33,34].

This paper presents the concept of the matrix description of material properties, e.g., constitutive relations. This concept is similar to that of Sussman and Bathe in one point— interpolation approach in both cases is used to ensure high consistency of constitutive relation with the experiment. The difference is that Sussman and Bathe used the interpolation of a one-variable function and the study uses the approximation of a two-variable function. The utilization of two-variable function is the most important difference between the presented solution and those used so far in other studies. The stress–strain (or moment–curvature for bending elements) relation is usually the basis for determining the material stiffness. The stiffness determined this way is a function of one independent variable, i.e., stress or strain. This paper proposes to treat stiffness as a function of two variables: stress and strain. This description of stiffness allows us to include more information about the material, e.g., partial history of previous strain or strain states.

The first part of this paper presents general assumptions for the description of stiffness using matrices and discusses the numerical algorithm to use stiffness matrices for analyses of the displacement and strain of a structure under load. The second part contains calculation examples and some possibilities of describing stiffness using a matrix are presented. The summary of the paper outlines the possible development of the presented concept and potential fields of its use.

The motivation for this paper was an interest in numerical methods and a desire to broaden their application scope in order to create alternative constitutive models of building materials. The main objective of this paper was to present the developed method for modeling the physical characteristics of materials. It is a discrete, matrix-described model. An appropriate numerical algorithm was developed to demonstrate the model effectiveness and some application examples. It was used in the presented calculation examples. The developed method of creating numerical constitutive models is alternative to classical, analytical approaches. No similar approach presented in this paper has been found in the scientific literature. Matrix modeling of constitutive features of materials has both advantages and limitations, compared to classical approaches. It has a potential for researchers involved in nonlinear structural analysis, e.g., made of hyper-elastic materials.

## 2. Materials and Methods

### 2.1. The Matrix Description of Material Stiffness Concept

In order to improve the commonly used approach to describe the properties of building materials, the concept of describing the coefficient of elasticity E as a function of two variables was developed, i.e., strain ε and stress σ. Different values of elasticity coefficients for load and unload may occur in a given state of the body defined by values (σ, ε), therefore the description of material stiffness requires two functions, which are indicated as $E^{p}$ and $E^{n}$. $E^{p}\left(\sigma ,\;\varepsilon \right)$ is a function of Young’s modulus at primary load cycle, $E^{n}\left(\sigma ,\;\varepsilon \right)$ is a function for the unload and secondary load cycle. These functions are related to tangential modules.

**Figure 1:**
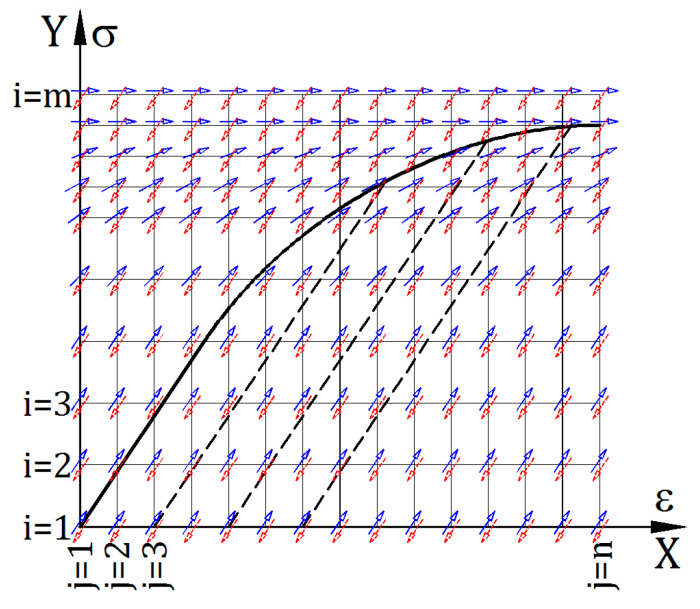
Graphical interpretation of the stiffness matrix structure.

Function E(σ, ε) is presented in the form of a discrete array of values that can be conveniently used in computer calculations. This value table is called the stiffness matrix in this paper. The graphical representation of the idea of such a matrix structure is presented in Figure 1.

In Figure 1, both the X axis (ε values) and Y axis (σ values) were digitized, but the division does not have to be uniform. X-axis values are collected in the X vector:

(1) $$X=\left[\varepsilon_{1},\varepsilon_{2},\ldots ,{\;\varepsilon }_{j},\ldots ,{\;\varepsilon }_{n}\right]$$

and Y-axis values—in the Y vector:

(2) $$Y=\left[\sigma_{1},\sigma_{2},\ldots ,\;\sigma_{i},\ldots ,\;\sigma_{m}\right]$$

The graphical interpretation of Young’s tangential moduli for the primary load cycle is shown with solid blue arrows pointing upwards, and for the unload and secondary load cycles—with red dashed arrows pointing downwards. Arrow directions are tangential to possible σ(ε) graphs. The general form of stiffness matrices is the following:

(3) $$E^{p}\left(\sigma ,\;\varepsilon \right)=\left[\begin{array}{ccccc} E_{11}^{p} & \cdots & E_{1j}^{p} & \cdots & E_{1n}^{p}\\ \vdots & \ddots & \vdots & & \vdots \\ E_{i1}^{p} & \cdots & E_{\mathrm{ij}}^{p} & \cdots & E_{\mathrm{in}}^{p}\\ \vdots & & \vdots & \ddots & \vdots \\ E_{m1}^{p} & \cdots & E_{\mathrm{mj}}^{p} & \cdots & E_{\mathrm{mn}}^{p} \end{array}\right],$$

(4) $$E^{n}\left(\sigma ,\;\varepsilon \right)=\left[\begin{array}{ccccc} E_{11}^{n} & \cdots & E_{1j}^{n} & \cdots & E_{1n}^{n}\\ \vdots & \ddots & \vdots & & \vdots \\ E_{i1}^{n} & \cdots & E_{\mathrm{ij}}^{n} & \cdots & E_{\mathrm{in}}^{n}\\ \vdots & & \vdots & \ddots & \vdots \\ E_{m1}^{n} & \cdots & E_{\mathrm{mj}}^{n} & \cdots & E_{\mathrm{mn}}^{n} \end{array}\right].$$

Matrix values describe the following relations:

(5) $$E_{\mathrm{ij}}^{p}=\mathrm{tg}\left(\alpha_{\mathrm{ij}}^{p}\right),$$

(6) $$E_{\mathrm{ij}}^{n}=\mathrm{tg}\left(\alpha_{\mathrm{ij}}^{n}\right).$$

**Figure 2:**
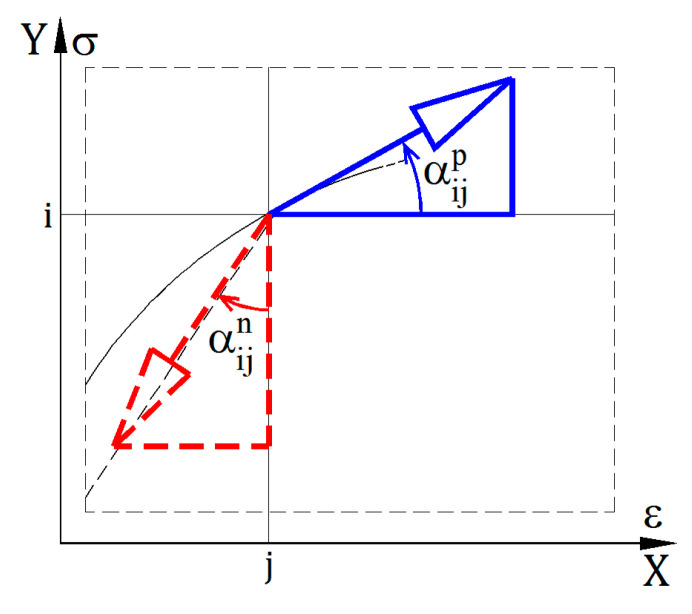
$\alpha_{\mathrm{ij}}^{p}$ and $\alpha_{\mathrm{ij}}^{n}$ angles at point $\left(\sigma_{i},{\;\varepsilon }_{j}\right)$.

The $\alpha_{\mathrm{ij}}^{p}$ and $\alpha_{\mathrm{ij}}^{n}$ angles are shown in Figure 2, that is a fragment of the graph presented in Figure 1.

Stiffness matrices created according to discussed principles are used later in this paper.

### 2.2. Calculation Algorithm That Utilizes Stiffness Matrices

Stiffness matrices contain tabular values of tangential moduli and are therefore suitable for use primarily in incremental methods of solving non-linear issues. In this case, two basic calculation problems arise. The first one is to determine the value of the Young modulus at any point outside the nodes. The second problem is related to the determination of the modulus value in a given calculation step (including increment).

**Figure 3:**
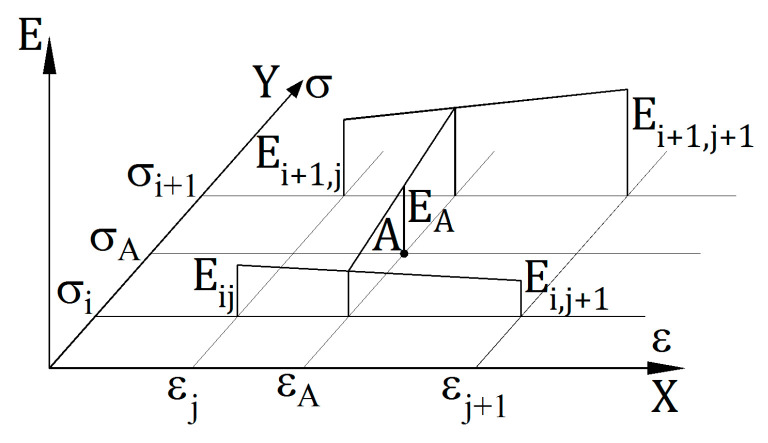
Determining the modulus value at point A.

A node should be understood as a point defined with a pair of values $\left(\sigma_{i},{\;\varepsilon }_{j}\right)$ that occur in vectors Y and X for which the modulus value $E_{\mathrm{ij}}$ is given in the stiffness matrices. In general, it is necessary to set modulus values for any points. For a selected A point, defined with a pair of values $\left(\sigma_{A},{\;\varepsilon }_{A}\right)$, the determination of the modulus value $E_{A}$ requires to run the algorithm in two steps. The first step is to establish the value of vectors Y and X between which the point A lies. This step is just to determine such values i and j that $\sigma_{i}\leq \sigma_{A}\leq \sigma_{i+1}$ and $\varepsilon_{j}\leq \varepsilon_{A}\leq \varepsilon_{j+1}$. The second step is to determine the modulus value at the A point. The principle is shown in Figure 3.

Taking into account the markings shown in Figure 3 and additionally defining:

(7) $$\Delta \sigma_{i}=\sigma_{i+1}-\sigma_{i},\;\Delta \varepsilon_{j}=\varepsilon_{j+1}-\varepsilon_{j},\;\Delta \sigma_{A}=\sigma_{A}-\sigma_{i},\;\Delta \varepsilon_{A}=\varepsilon_{A}-\varepsilon_{j},$$

the requested value is determined with the following relation:

(8) $$\begin{array}{c} E_{A}=E_{\mathrm{ij}}\left(1-\frac{\Delta \sigma_{A}}{\Delta \sigma_{i}}\right)\left(1-\frac{\Delta \varepsilon_{A}}{\Delta \varepsilon_{j}}\right)+E_{i,j+1}\left(1-\frac{\Delta \sigma_{A}}{\Delta \sigma_{i}}\right)\frac{\Delta \varepsilon_{A}}{\Delta \varepsilon_{j}}+E_{i+1,j}\;\frac{\Delta \sigma_{A}}{\Delta \sigma_{i}}\left(1-\frac{\Delta \varepsilon_{A}}{\Delta \varepsilon_{j}}\right)+\\ E_{i+1,j+1}\frac{\Delta \sigma_{A}}{\Delta \sigma_{i}}\;\frac{\Delta \varepsilon_{A}}{\Delta \varepsilon_{j}}. \end{array}$$

As presented in Figure 3 and Formula (8), linear interpolation was used to determine the modulus value outside the nodes.

**Figure 4:**
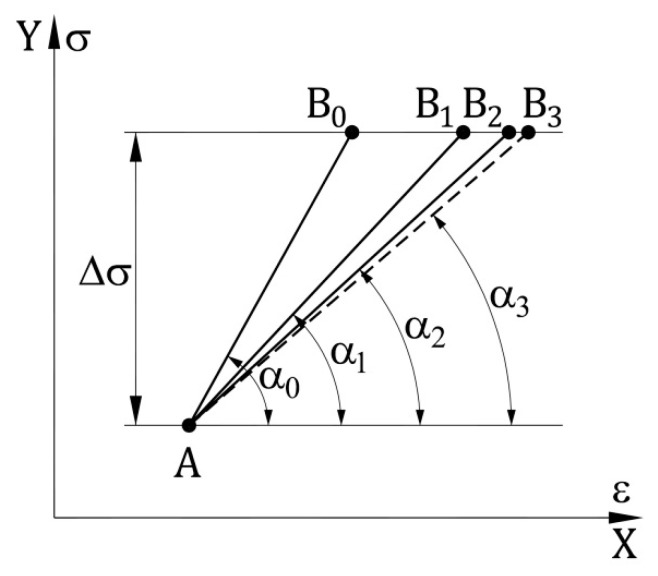
Determining the modulus value in one calculation step.

When determining the elasticity modulus in a given calculation step, it is assumed that the value of stress increase Δσ is known. The determination of its corresponding Δε value, and thus the value of Young’s modulus in a given step, is done with successive approximations. The idea of this process is presented in Figure 4.

Let us assume that stiffness matrix, point A coordinates and increment value Δσ are given. The value of the stiffness modulus at the A point shall be determined according to Formula (8). During the first, initial approximation, angle $\alpha_{0}$ is assumed so that $\mathrm{tg}\left(\alpha_{0}\right)=E_{A}$ and the position of the $B_{0}$ point is calculated. Then, with a second approximation, the $E_{B0}$ modulus value is determined (i.e., value at the $B_{0}$ point) and the $\alpha_{1}$ calculated angle is that $\mathrm{tg}\left(\alpha_{1}\right)=0.5\left(E_{A}+E_{B0}\right)$. This enables the calculation of the position of the $B_{1}$ point and with similar actions, as in case of $\alpha_{1}$, determine the $\alpha_{2}$ angle. Such calculations are performed to achieve assumed α angle accuracy and thus the accuracy of the Young’s modulus. The value of the modulus can be determined in the same way if the strain increase Δε in one step is known.

The basic elements of a calculation algorithm to use the stiffness matrix are very simple, and based on elementary mathematical operations. However, the algorithm simplicity should be considered as its advantage.

### 2.3. Flowchart of the Calculation Algorithm

**Figure 5:**
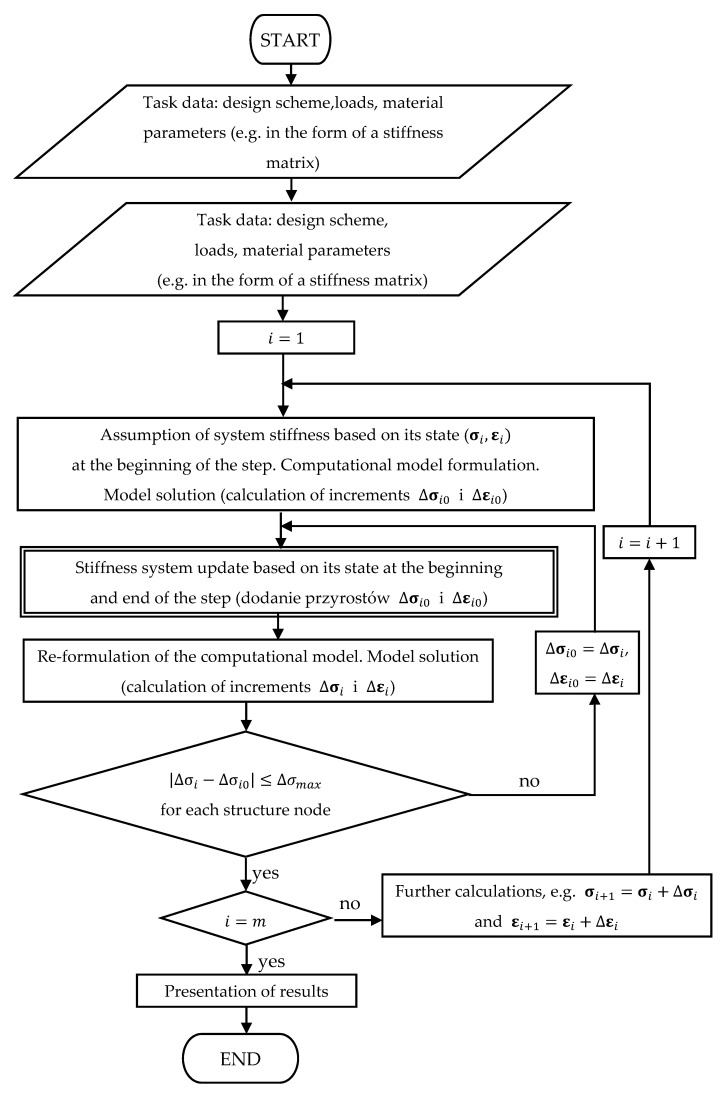
A flowchart of the calculation algorithm.

**Figure 6:**
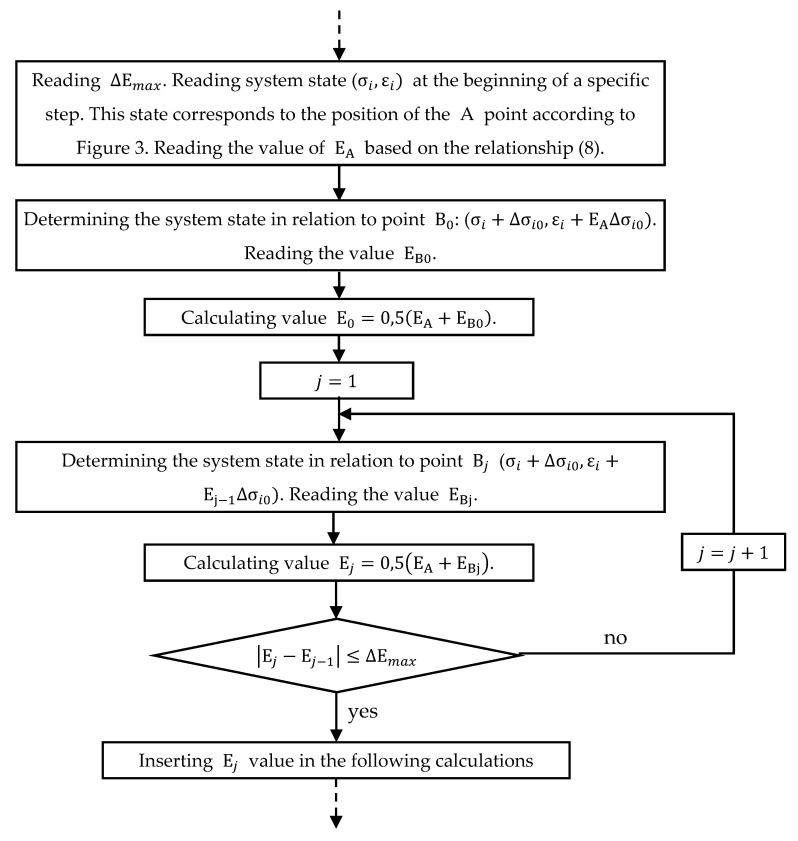
A flowchart that presents the stiffness analysis within the main algorithm.

Figure 5 shows a general basic block diagram of the calculation algorithm. It is a simplified diagram that focuses on activities relevant to the issues at hand. The activities performed in the operation block marked with a double box are shown in Figure 6. These refer to the issues described in Section 2.2.

For each node of the discretized structure:

## 3. Results

The first part was limited to the presentation of simulation examples of relatively simple sample tests made of an abstract elastic–plastic material. These tests are the axial tensile stress bar test, cyclic tensile and relief stress bar test, and the tensile stress bar test with relief, compression and another relief stage. During the second part, the tension of three bars connected with a rigid beam was simulated and the reinforced concrete beam was calculated.

For the purpose of the presented simulations, a hypothetical material was adopted, the Young’s modulus of which is described with the values of dimensionless deformations ε collected in the X vector:

(9) $$X=\left[\begin{array}{cccccc} 0.00 & 0.04 & 0.08 & 0.12 & 0.16 & 0.20 \end{array}\right].$$

σ stress values expressed in (MPa), collected in the Y vector:

(10) $$Y=\left[\begin{array}{cccccccccc} 0.00 & 1.22 & 2.44 & 3.67 & 4.89 & 6.11 & 7.33 & 8.56 & 9.78 & 11.00 \end{array}\right]$$

and two stiffness matrices, expressed in (MPa):

(11) $$E^{p}={\left[\begin{array}{cccccccccc} 200 & 200 & 195 & 185 & 163 & 135 & 100 & 50 & 20 & 0\\ 200 & 200 & 195 & 185 & 163 & 135 & 100 & 50 & 20 & 0\\ 200 & 200 & 195 & 185 & 163 & 135 & 100 & 50 & 20 & 0\\ 200 & 200 & 195 & 185 & 163 & 135 & 100 & 50 & 20 & 0\\ 200 & 200 & 195 & 185 & 163 & 135 & 100 & 50 & 20 & 0\\ 200 & 200 & 195 & 185 & 163 & 135 & 100 & 50 & 20 & 0 \end{array}\right]}^{T},$$

(12) $$E^{n}={\left[\begin{array}{cccccccccc} 200 & 200 & 200 & 200 & 200 & 200 & 200 & 200 & 200 & 200\\ 200 & 200 & 200 & 200 & 200 & 200 & 200 & 200 & 200 & 200\\ 200 & 200 & 200 & 200 & 200 & 200 & 200 & 200 & 200 & 200\\ 200 & 200 & 200 & 200 & 200 & 200 & 200 & 200 & 200 & 200\\ 200 & 200 & 200 & 200 & 200 & 200 & 200 & 200 & 200 & 200\\ 200 & 200 & 200 & 200 & 200 & 200 & 200 & 200 & 200 & 200 \end{array}\right]}^{T}.$$

### 3.1. Example A. Axial Bar Tension

The first analyzed issue is relatively simple. Its solution is to be used primarily to study the course of the σ(ε) function. It is clear that in case of analyzing real issues, laboratory tests must first be performed to identify the σ(ε) relation, and then appropriate stiffness matrices $E^{p}$ and $E^{n}$ must be created. However, stiffness matrices themselves and graphs based on them are not directly represented in the σ(ε) relation. Only the laboratory sample simulation, e.g., the axial bar tension, will allow the corresponding σ(ε) graph to be depicted.

It is assumed that a bar that is stretched has a length L = 2.0 m and a cross-section area A = 0.01 m^2^. The bar material is described with matrices (11) and (12). Tension is static and continues until a tensile force of 105kN is reached.

To solve the issue of the tensile bar simulation, the algorithm discussed in the previous chapter was used. The algorithm was the basis for a computer program written in the Scilab environment. The selection of the Δσ step (see Figure 4) was carried out adaptively so that the transition of the graph through one cell of the stiffness matrix (the field separated with two adjacent values from the X vector and two adjacent values from the Y vector) took place in about four steps.

**Figure 7:**
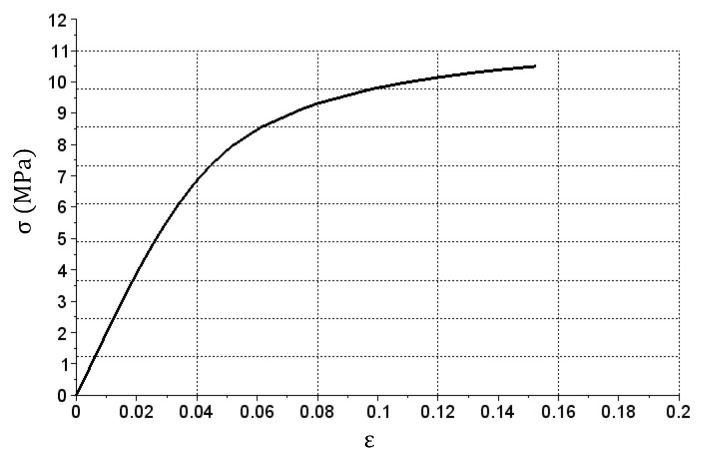
Diagram of σ(ε) relation obtained after performing bar tension simulation.

The graph based on the simulation is shown in Figure 7. Since a continuous stress increase was present during the simulation, only the $E^{p}$ matrix was used in the calculations.

The graph presented in Figure 7 shows the material behavior, described with X vectors according to (9), Y vectors according to (10) and the $E^{p}$ matrix given in (11).

Based on experimental data, it is quite simple to create a suitable stiffness matrix. This will be presented in the next example.

### 3.2. Example B. Stiffness Matrix Calibration with Axial Tensile Bar Test Simulation

- Case 1

The following example shows the results of stiffness matrix calibration. It was created manually by introducing subsequent corrections in the matrix values and observing the obtained effects. Let us assume that the tensile bar test produced the F(ΔL) relation graph. Some characteristic points are indicated in this graph and then the σ stresses corresponding to the F forces and the ε strains corresponding to ΔL elongations are calculated for these points. Finally, the σ(ε) graph should go through the vector points

(13) $$\hat{\varepsilon }=\left[0\;\;0.002\;\;0.0022\;\;0.0031\;\;0.004\;\;0.006\;\;0.009\;\;0.014\right],$$

and

(14) $$\hat{\sigma }=\left[00.580.600.6050.610.750.850.90\right].$$

Values in the $\hat{\varepsilon }$ vector are dimensionless and values in the $\hat{\sigma }$ vector are expressed in (MPa). Additionally, the graph in the first section (between points 1 and 2) should be linear.

The vectors X, Y and the $E^{p}$ matrix were produced with successive value adjustments to finally obtain the following:

(15) X=[0  0.0010  0.0020  0.0022  0.0031  0.0040  0.0041  0.0060  0.0090  0.0140  0.0160],

(16) Y=[0  0.29  0.58  0.60  0.605  0.61  0.615  0.75  0.80  0.85  0.90  0.95],

(17) $$E_{11\times 12}^{p}={\left[\begin{array}{cccccccccccc} 290 & 290 & 290 & 5.6 & 5.6 & 5.6 & 77 & 70 & 37 & 15 & 6.3 & 0\\ 290 & 290 & 290 & 5.6 & 5.6 & 5.6 & 77 & 70 & 37 & 15 & 6.3 & 0\\ & & & & & \vdots & & & & & & \\ 290 & 290 & 290 & 5.6 & 5.6 & 5.6 & 77 & 70 & 37 & 15 & 6.3 & 0 \end{array}\right]}^{T}.$$

**Figure 8:**
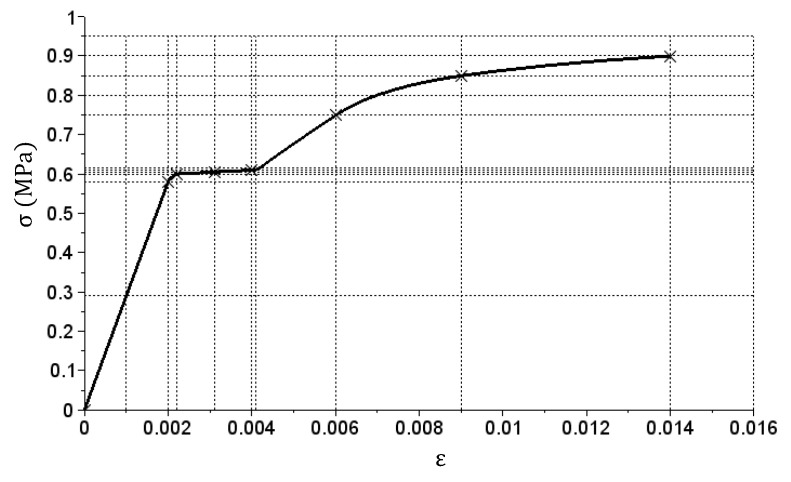
σ(ε) relation graph achieved for the calibrated stiffness matrix in case 1.

Values in the X vector are dimensionless, and values in the Y vector and the $E^{p}$ matrix are expressed in (MPa). For a specific material stiffness determined with this method, the axial tensile test was simulated until a stress value of 0.9 MPa was reached. A computer program created for example A was used. Figure 8 shows an achieved tensile simulation graph for the calibrated stiffness matrix (solid line). Points defined with $\hat{\varepsilon }$ and $\hat{\sigma }$ vectors are marked with crosses. It can be seen that the tensile simulation graph matches the base points quite well. The shape of the resulting graph corresponds to a material with a distinct yield strength, e.g., low-carbon steel.

- Case 2

In the second example, the same actions were performed as before, assuming the following set of base data:

(18) $$\hat{\varepsilon }=\left[0\;\;0.001\;\;0.0016\;\;0.0022\;\;0.0030\;\;0.0040\;\;0.0051\;\;0.0063\;\;0.0074\;\;0.0082\;\;0.0090\right],$$

(19) $$\hat{\sigma }=\left[00.21\;\;0.29\;\;0.33\;\;0.36\;\;0.38\;\;0.40\;\;0.44\;\;0.52\;\;0.63\;\;0.80\right].$$

These are the experimental data read from Figure 6b in the paper [23].

In this case, stiffness is described with the following vectors

(20) $$X=\left[\begin{array}{cccccccccccccccc} 1 & 1.02 & 1.06 & 1.11 & 1.2 & 1.31 & 1.42 & 1.68 & 1.94 & 2.49 & 3.03 & 3.43 & 3.75 & 4.07 & 4.26 & 4.45 \end{array}\right],$$

(21) $$Y=\left[\begin{array}{cccccccccccccccc} 0 & 0.1 & 0.15 & 0.24 & 0.33 & 0.44 & 0.51 & 0.69 & 0.77 & 0.96 & 1.24 & 1.44 & 1.71 & 1.97 & 2.2 & 2.42 \end{array}\right],$$

and the matrix

(22) $$E_{16\times 16}^{p}={\left[\begin{array}{cccccccccccccccc} 7 & 2 & 1.6 & 1.5 & 1.1 & 0.9 & 0.65 & 0.35 & 0.38 & 0.4 & 0.53 & 0.65 & 0.85 & 1.05 & 1.12 & 1.3\\ 7 & 2 & 1.6 & 1.5 & 1.1 & 0.9 & 0.65 & 0.35 & 0.38 & 0.4 & 0.53 & 0.65 & 0.85 & 1.05 & 1.12 & 1.3\\ & & & & & & & \vdots & & & & & & & & \\ 7 & 2 & 1.6 & 1.5 & 1.1 & 0.9 & 0.65 & 0.35 & 0.38 & 0.4 & 0.53 & 0.65 & 0.85 & 1.05 & 1.12 & 1.3 \end{array}\right]}^{T}.$$

**Figure 9:**
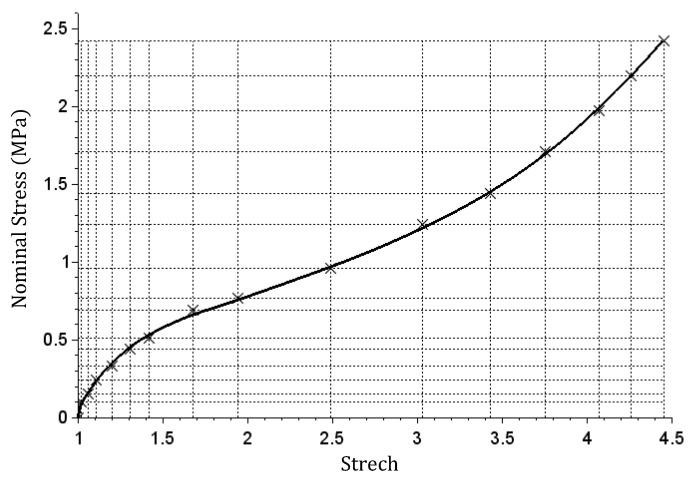
Relation graph achieved for the calibrated stiffness matrix in case 2 (the axis designations used in Figure 6b in Ref. [23] were used).

The graph shown in Figure 9 was obtained with simulating the tensile test. A high degree of compatibility with the base data was also achieved. The shape of the graph is similar to the ones corresponding to hyper-elastic materials that offer a stiffness increase at high deformations. Rubber is a good example of such material.

In both examples, material stiffness matrices were developed to reproduce hypothetical laboratory tensile sample tests of different materials with high accuracy (evaluated visually, based on graphs). It should be noted that there were different increments between the values of X and Y vectors. As a consequence, the Δσ increments used in the algorithm are adjusted accordingly. This means that the value density in X and Y vectors in the zones with more rapid changes in the elasticity modulus automatically caused a decrease in Δσ step and thus better algorithm accuracy.

In both cases, the values in vectors X and Y are mostly repetitions of the base data set in vectors $\hat{\varepsilon }$ and $\hat{\sigma }$. Such a relationship is fairly obvious, but not a requirement. Selection of values in vectors $\hat{\varepsilon }$ and $\hat{\sigma }$ depends on the person that builds the stiffness matrix of a given material. It must be done in such a way that it closely reproduces the tension graph obtained in the laboratory. In zones of large changes of the inclination angle that is tangent to the graph, the value drops in vectors $\hat{\varepsilon }$ and $\hat{\sigma }$ have to be small—this is clearly visible in Figure 8. Incorrect selection of base values in vectors $\hat{\varepsilon }$ and $\hat{\sigma }$ will lead to the creation of an inadequate constitutive model. A solution to the problem would also be to use small increments in complete vectors $\hat{\varepsilon }$ and $\hat{\sigma }$, but it would lead to a significant size increase of the stiffness matrix.

### 3.3. Example C. Axial Cyclic Tension and Relief Stress Bar Test

**Figure 10:**
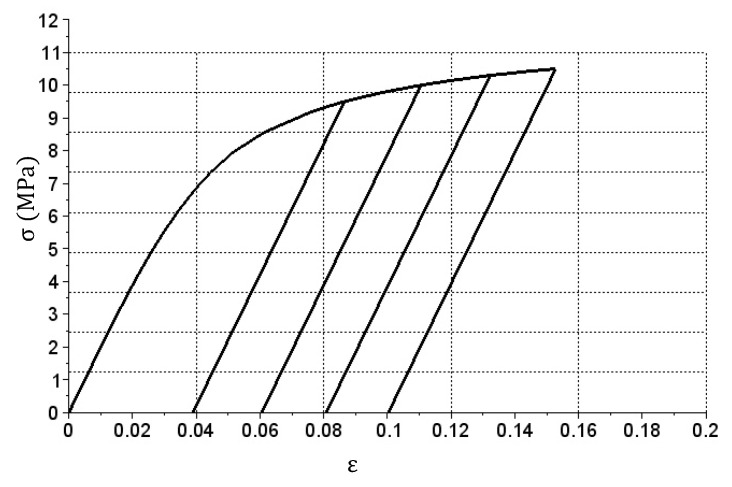
σ(ε) relation graph for the simulation of cyclic tension and relief of the bar.

This example shows a simulation of the tension and relief stress test of the bar. The subject of the analysis will be exactly the same bar that is made of the same material as in example A (Section 3.1). This time, however, it was assumed that the bar would be cyclically stretched to a certain force value and then relieved. During individual cycles, tensile forces of 95, 100, 103 and 105 kN, respectively, were assumed to be achieved. The obtained tension graph is shown in Figure 10.

In this case, only the $E^{p}$ matrix was used during the first load cycle. During the relief and another load stage to the previously achieved stress level, the values were taken from the $E^{n}$ matrix. When applying load above the previously achieved stress level, the $E^{p}$ matrix was reused.

**Figure 11:**
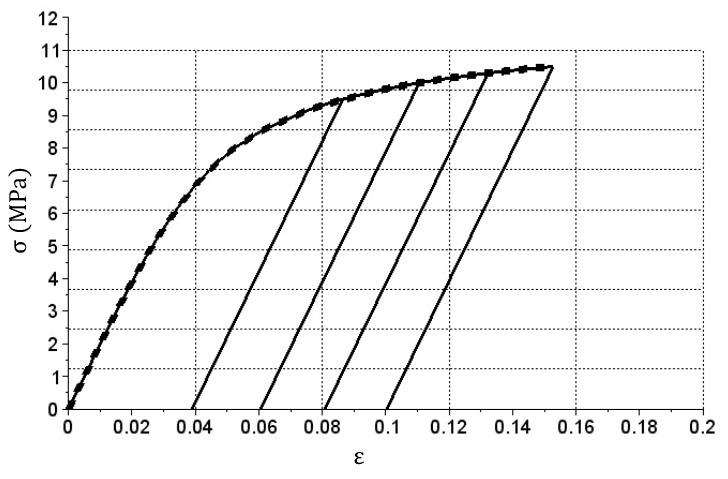
Comparison of the cyclic tension and relief graph (solid thin line) with a single cycle tension graph (dashed thick line).

The graph obtained in Figure 10 corresponds to the model behavior of such materials as steel. Extreme upper parts of the graph from Figure 10 should match the graph presented in Figure 7. The confirmation of this rule was obtained by comparing both graphs in Figure 11.

**Figure 12:**
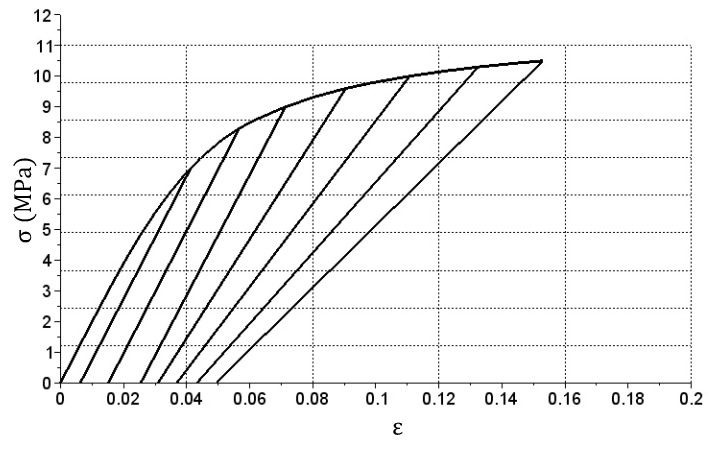
σ(ε) relation graph produced during the simulation of cyclic tension and relief of the bar with changing $E^{n}$ matrix values.

The graph shown in Figure 10 shows that the lines corresponding to reliefs and consecutive repeated loads in successive cycles are parallel to one another. This is obvious, as only the $E^{n}$ matrix with constant coefficients is used in the simulation during these phases. Appropriate calibration of this matrix may result in a change of the slope in graph sections related to relief and another application of load. Another method is to change $E^{n}$ stiffness matrix coefficients during the calculation. Figure 12 shows an example graph of cyclic tension and relief with such matrix modification.

In the presented example, after the sample material exceeded certain deformation limits (0.07 in this case), the matrix was multiplied by $E^{n}$, by a coefficient whose value was a certain function of achieved deformation. Such a mechanism can be used, for example, to build degradation models.

### 3.4. Example D. Sample Tension and Compression—Hysteretic Material Response

This example shows the simulation of a tensile sample test described in example A (Section 3.1) with a force of 107 kN, followed by a relief, compression with a force of 107 kN and final relief. In order to exclude the possibility of stability loss by the compressed sample, this time it was assumed that the sample length will be 0.2 m.

**Figure 13:**
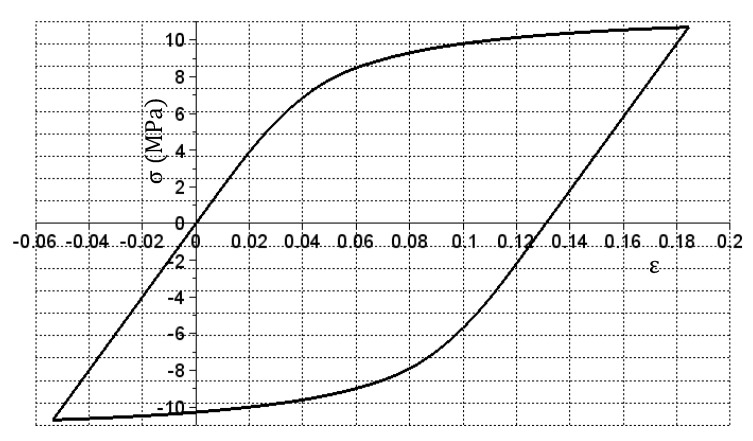
The σ(ε) relation obtained during the tension and subsequent compression sample simulation.

The simulation required to extend the stiffness matrix. First, the vectors were expanded: X with the relation (9) and Y with the relation (10) that add specific values symmetrically to the 0.00 values and then change their signs to negative. In case of the $E^{p}$ matrix given with a Formula (11), symmetrization relative to the first column (containing values of 200) was performed and an appropriate number of repeating rows was also added. The $E^{n}$ matrix has been expanded to appropriate size and filled with 200 values (see relation (12)). For the data prepared in this way, a simulation was performed that produced the σ(ε) relation graph shown in Figure 13.

As it can be seen in Figure 13, a hysteresis loop graph was obtained. The shape results from the adoption of the symmetry of values in the $E^{p}$ matrix. Precise loop closure at the (0.0) point additionally indicates high accuracy of the algorithm used.

### 3.5. Example E. Tension and Relief of the Three Bar System

**Figure 14:**
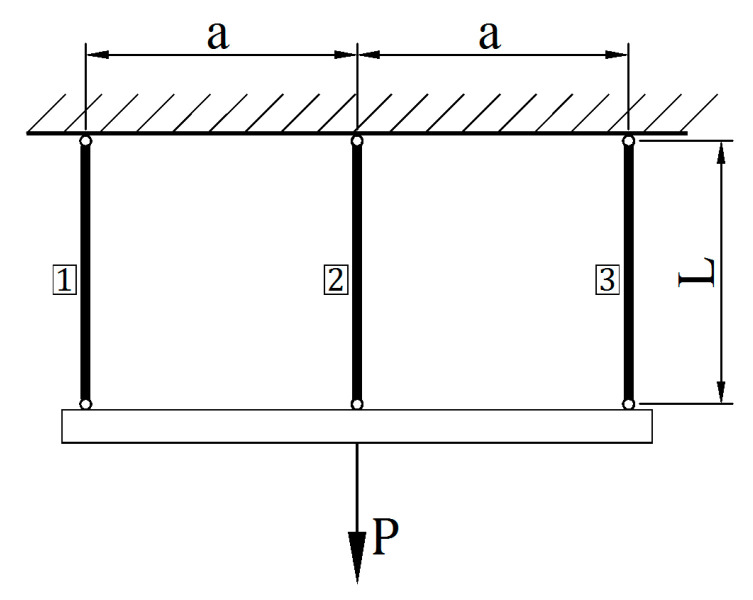
A three bar system in tension.

The next analyzed issue is related to the tension of the system of three connected bars shown in Figure 14.

The bars have the same length L and the distances between them are constant and are indicated as a. It is assumed that the upper fastening position of each bar (marked with numbers as i=1, 2 and 3) are not displaced in any way. At the bottom, the bars are attached to a beam, which was treated as a rigid body. The tensile force P is transmitted to the system through the beam. Individual bars have different cross-section areas $A_{i}$ and can also be made of materials that feature different properties. Tensile forces inside the bars are indicated as $P_{i}$.

The system being solved is statically indeterminable. Three equations have been formulated to solve the problem: two static equations and one displacement-related equation, resulting from the assumption that the beam is a rigid body (no deformations). The sum of forces equation of the analyzed case is as follows:

(23) $$P_{1}+P_{2}+P_{3}=P.$$

The equation of the sum of force moments related to any point on bar no. 2 results in the following relation:

(24) $$P_{1}-P_{3}=0.$$

The last relation on the assumed geometry of the system is in the form of the following:

(25) $$u_{2}=\frac{u_{1}+u_{3}}{2}\;\mathrm{or}\;u_{1}-2u_{2}+u_{3}=0,$$

where $u_{i}$ indicates the vertical displacement of the no. i bar end, that is elongation of this bar. Introducing the relation describing the classical Hooke’s law ($P_{i}=E_{i}A_{i}u_{i}/L$) into the above equations, a system of three equations with unknown $u_{i}$ displacements is obtained:

(26) $$\left[\begin{array}{ccc} E_{1}A_{1}/L & E_{2}A_{2}/L & E_{3}A_{3}/L\\ E_{1}A_{1}/L & 0 & -E_{3}A_{3}/L\\ 1 & -2 & 1 \end{array}\right]\left[\begin{array}{c} u_{1}\\ u_{2}\\ u_{2} \end{array}\right]=\left[\begin{array}{c} P\\ 0\\ 0 \end{array}\right].$$

Assuming that $E_{i}=f\left(\sigma_{i},{\;\varepsilon }_{i}\right)$, and thus $E_{i}=f\left(u_{i}\right)$, the above system is non-linear.

As in previous examples, the equation system was solved using the incremental method with a previously described computational algorithm implemented in the proprietary program created in the Scilab environment. The stiffness of the bar material is described with $E_{i}^{p}$ and $E_{i}^{n}$ matrices. For rods No. 1 and 3 the stiffness matrices identical to problem D were used, while in case of rod No. 2 the matrices from problem D were multiplied by 0.75.

A cycle consisting of a load phase until the P force reaches 3.5 MN and a load relief phase were assumed. The following length L = 2.0 m and bar cross-section areas are assumed: $A_{1}$ = 0.17 m^2^, $A_{2}$ = 0.20 m^2^, $A_{3}$ = 0.09 m^2^. It was assumed that the whole system is weightless and that there were no stresses in the bars before the start of applying the load.

**Figure 15:**
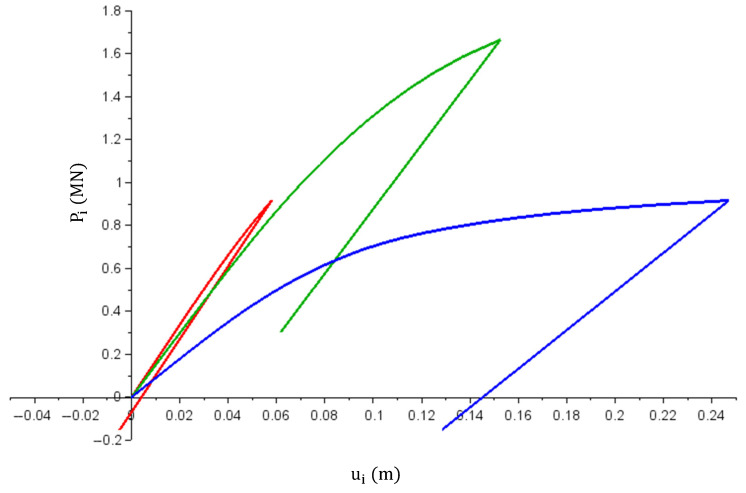
$P_{i}\left(u_{i}\right)$ graph (i = 1—red; i = 2—green; i = 3—blue).

**Figure 16:**
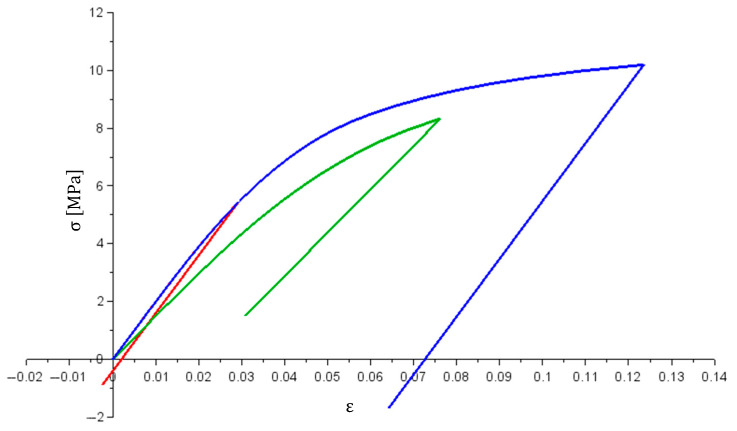
$\sigma_{i}\left(\varepsilon_{i}\right)$ graph (i = 1—red; i = 2—green; i = 3—blue).

Calculation results are shown in Figure 15 and Figure 16. Figure 15 shows the relations between the $P_{i}$ forces (MN) in specific bars and their elongations $u_{i}$ (m). The graphs are color coded: red for bar No. 1, green for bar No. 2 and blue for bar No. 3. The combined maximum forces achieved with individual bars reach a value of 3.5 MN. After load relief, there are forces in the bars, the sum of which is 0. Bars No. 2 and No. 3 were extended from their original length and bar No. 1 was shortened.

Graphs in Figure 16 show the relation between $\sigma_{i}$ stresses [MPa] and $\varepsilon_{i}$ strains. It can be seen that the load curves for bars no. 1 no. 3 overlap (partly as more stress is achieved in bar no. 3), while the curve for bar no. 2 is more inclined. This is also the case during the relief stage. It is caused by different stiffness of the materials from which the bars are made.

The presented example differs from the previous ones as its solution required solving a system of non-linear equations. Based on the achieved results, it was concluded that:

- Obtained solutions (in each calculation step) met the equations of the solved equation system;
- $\sigma_{1}\left(\varepsilon_{1}\right)$ and $\sigma_{3}\left(\varepsilon_{3}\right)$ stress graphs have the same shape as the graph in Figure 7 (stiffness matrices are the same in each case); a similar check was also performed for the $\sigma_{2}\left(\varepsilon_{2}\right)$ graph, and in this case, the charts were also confirmed to be consistent.

Indicated observations prove the correctness of the algorithm and the usefulness of the stiffness matrix for solving non-linear systems that are statically indeterminable.

### 3.6. Example F. Bending of a Reinforced Concrete Beam

**Figure 17:**
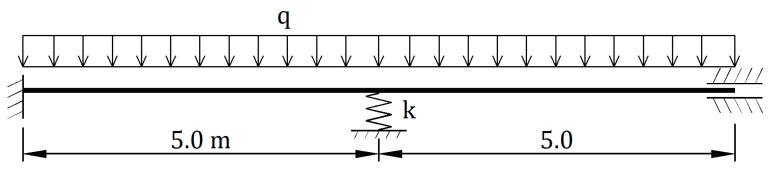
Static diagram of the beam being analyzed.

The last example is the most complex and, at the same time, features the most reference for practical aspects. It refers to bending of a double-span reinforced concrete beam shown in Figure 17. The beam cross section is rectangular in shape with the following dimensions b×h = 40.0 cm × 60.0 cm. The bottom reinforcement of 3 Ø20 in positive moment zones, and the top reinforcement of 8 Ø20 in negative moment zones were assumed. C30/37 concrete and 34GS reinforcing steel were used, and concrete features were taken from standard [35] Distances between reinforcement centers of gravity and beam edges are 4.0 cm. The stiffness of intermediate elastic support is k = 700 MN/m.

In this case, the stiffness matrices of two considered beam cross-sections (with different reinforcement) were built first. The algorithm for determining the stiffness of reinforced concrete bend cross-sections was used, which was defined in the paper [36]. It uses cross-sectional dimensions, surface area and location of main reinforcement, as well as the physical relations for concrete and reinforcing steel as defined in Eurocode 2. Based on achieved results, stiffness matrices were defined. Section stiffness with the reinforcement of 3 Ø20 is described with vectors

(27) $$X_{1}=\left[0\;\;1.22\;\;2.44\;\;3.65\;\;4.38\;\;7.06\;\;9.50\;\;12.18\;\;14.61\;\;17.05\;\;25.00\right]\times 10^{-3},$$

(28) $$Y_{1}=\left[0\;\;53.5\;\;106.7\;\;159.5\;\;191.0\;\;203.8\;\;205.3\;\;206.5\;\;207.2\;\;207.8\;\;208.3\;\;250.0\right]$$

and the matrix

(29) $$E_{1\;\left(11\times 13\right)}^{p}={\left[\begin{array}{ccccccccccccc} 44135 & 43854 & 43606 & 43186 & 41405 & 650 & 450 & 200 & 200 & 200 & 200 & 200 & 0\\ 44135 & 43854 & 43606 & 43186 & 41405 & 650 & 450 & 200 & 200 & 200 & 200 & 200 & 0\\ & & & & & \vdots & & & & & & & \\ 44135 & 43854 & 43606 & 43186 & 41405 & 650 & 450 & 200 & 200 & 200 & 200 & 200 & 0 \end{array}\right]}^{T}.$$

The $E_{1\;\left(11\times 13\right)}^{n}$ matrix consist entirely of 44,135 values.

Section stiffness with the reinforcement of 8 Ø20 is described with vectors

(30) $$X_{2}=\left[0\;\;1.00\;\;2.20\;\;3.40\;\;4.40\;\;5.40\;\;5.60\;\;7.80\;\;10.00\;\;12.20\;\;14.40\;\;16.60\;\;18.80\;\;31.70\right]\times 10^{-3},$$

(31) $$Y_{2}=\left[0\;\;95.3\;\;212.6\;\;327.8\;\;418.2\;\;506.9\;\;512.9\;\;521.1\;\;526.3\;\;529.9\;\;532.5\;\;534.5\;\;536.0\;\;537.5\;\;600.0\right]$$

and the matrix

(32) $$E_{2\;\left(14\times 15\right)}^{p}={\left[\begin{array}{cccccccccccccc} 98576 & 97360 & 95640 & 93719 & 92190 & 90424 & 8000 & 2200 & 1726 & 1449 & 800 & \cdots & 800 & 0\\ 98576 & 97360 & 95640 & 93719 & 92190 & 90424 & 8000 & 2200 & 1726 & 1449 & 800 & \cdots & 800 & 0\\ & & & & & & \vdots & & & & & & & \\ 98576 & 97360 & 95640 & 93719 & 92190 & 90424 & 8000 & 2200 & 1726 & 1449 & 800 & \cdots & 800 & 0 \end{array}\right]}^{T}.$$

The $E_{2\;\left(14\times 15\right)}^{n}$ matrix consist entirely of 98,576 values.

**Figure 18:**
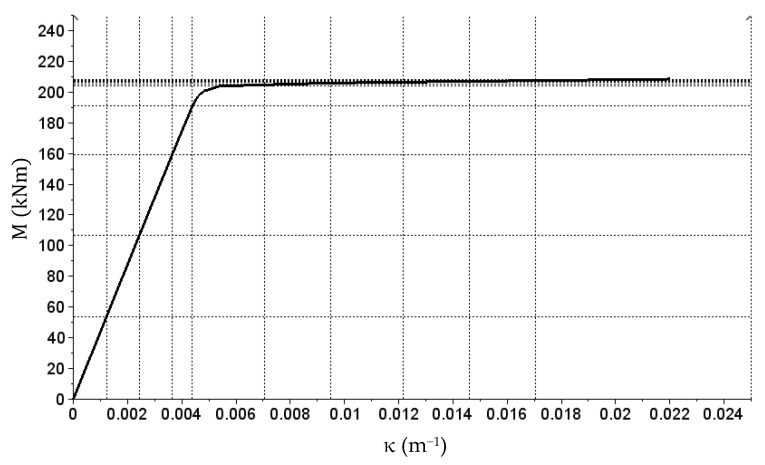
The M(κ) relation graph corresponding to the $E_{1}^{p}$ matrix (reinforcement of 3 Ø20).

**Figure 19:**
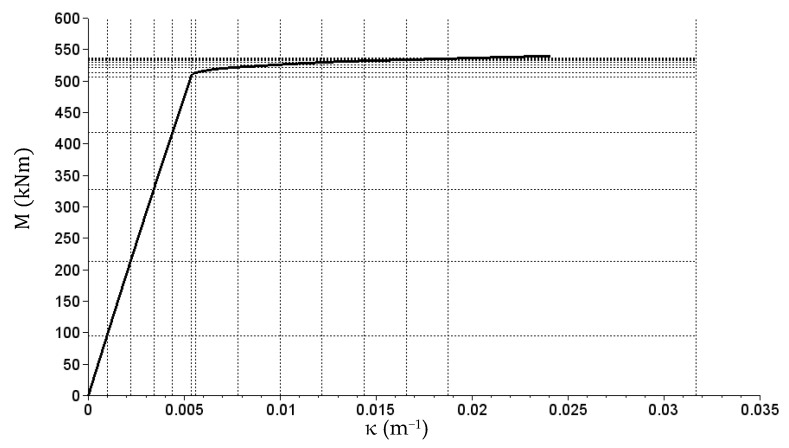
The M(κ) relation graph corresponding to the $E_{2}^{p}$ matrix (reinforcement of 8 Ø20).

Vectors $X_{1}$ and $X_{2}$ are curvature vectors here (κ) expressed in (m^−1^), $Y_{1}$ and $Y_{2}$ are bending moment vectors (M) expressed in (kNm), and matrices $E_{1\_}^{p}$, $E_{1}^{n}$, $E_{2\_}^{p}$ and $E_{2}^{n}$ describe the bending stiffness of sections expressed in (kNm^2^). The M(κ) relation graphs generated based on the data compiled above are presented in Figure 18 and Figure 19.

The problem was solved with the finite difference method and the incremental algorithm incorporating stiffness matrices that was discussed earlier and used in previous problems. The beam undergone discretization assuming a digitizing range of 0.1 m. One load cycle for the beam and the subsequent relief was assumed. Maximum load value of q = 160 kN/m was chosen so that the plasticization effect of beam sections could be clearly seen during the simulation. Building the load increment vector, the load phase was divided into two ranges. The first range, covering the force range from 0 to 0.95q (i.e., up to the value of 144 kN/m), was divided into 15 subranges with an increment of 9.60 kN/m. The second range, covering the force range from 0.95 q to q (i.e., from 144 to 160 kN/m), was divided into 15 subranges with an increment of 1.067 kN/m. The load phase (from the value q = 160 kN/m to 0) was divided into 15 subranges with an increment of 10.67 kN/m.

**Figure 20:**
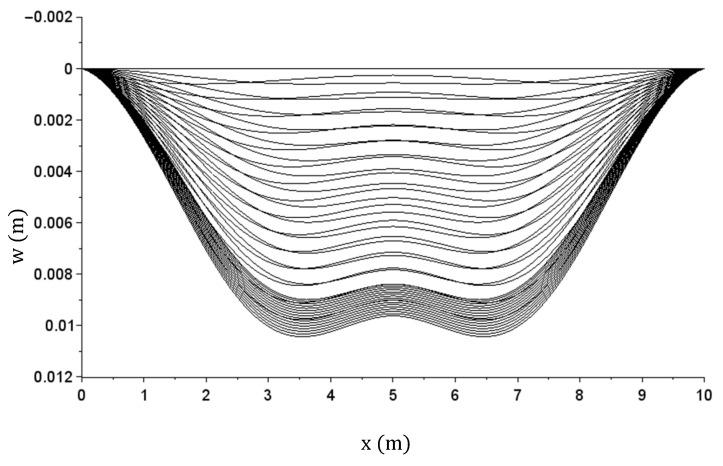
Beam deflection w(x) graph during specific load and relief steps.

The displacement (deflection) graph obtained during specific steps (for successive load values in the load and relief phases) is shown in Figure 20. The x axis describes the coordinates of the beam sections starting from the leftmost support.

**Figure 21:**
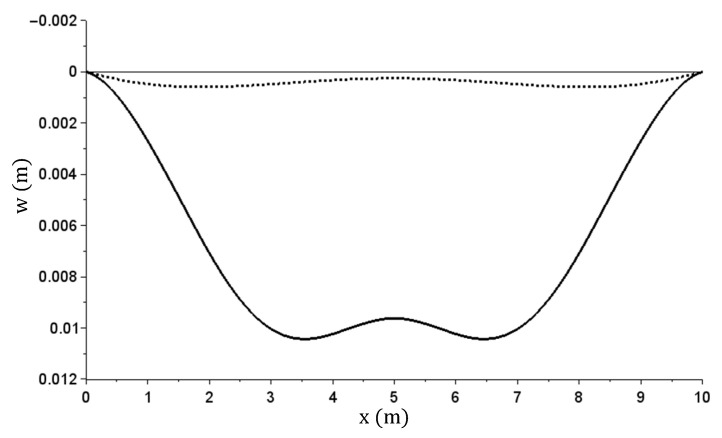
The w(x) [m] deflection graph—at maximum and after relief.

**Figure 22:**
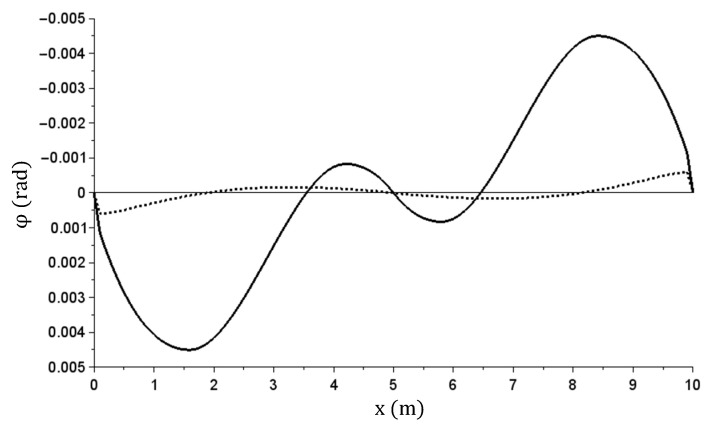
The φ(x) (rad) angle of rotation graph—at maximum and after relief.

**Figure 23:**
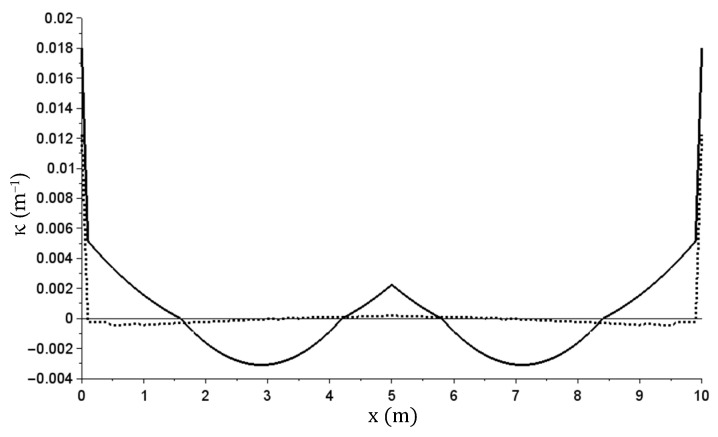
The κ(x) (m^−1^) curvature graph—at maximum and after relief.

**Figure 24:**
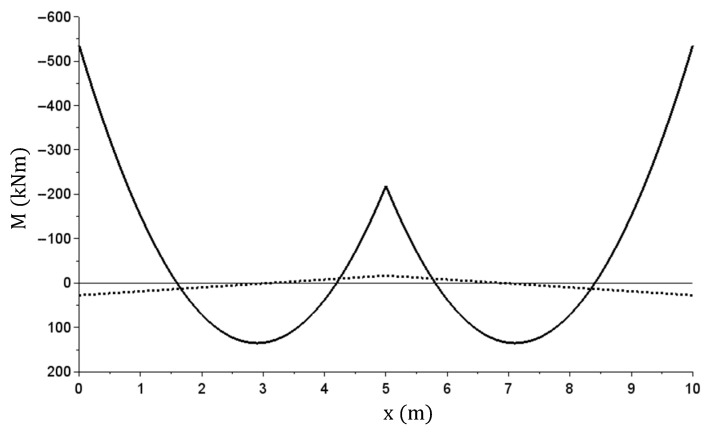
The M(x) (kNm) bending moment graph—at maximum and after relief.

**Figure 25:**
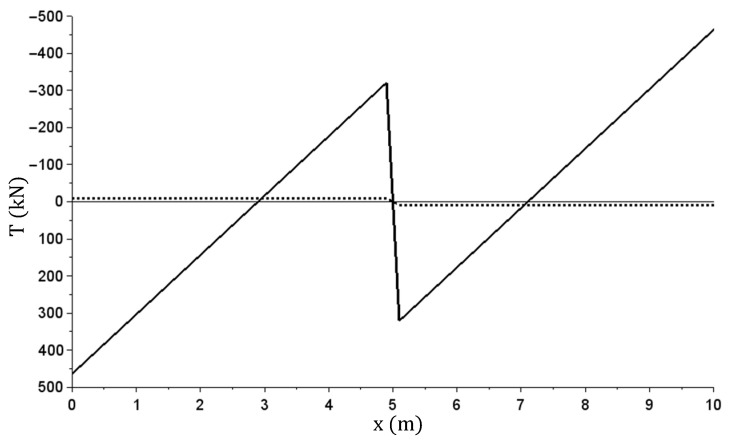
The T(x) (kN) shearing force graph—at maximum and after relief.

The other graphs (Figure 21, Figure 22, Figure 23Figure 24 and Figure 25) show only the calculation results corresponding to maximum load (q = 160 kN/m)—solid lines, and the total beam relief—dashed lines.

From the achieved results, it can be observed that the beam did not return to its original shape after load relief. The system is statically indeterminable and therefore non-zero internal forces in the beam also remained.

**Figure 26:**
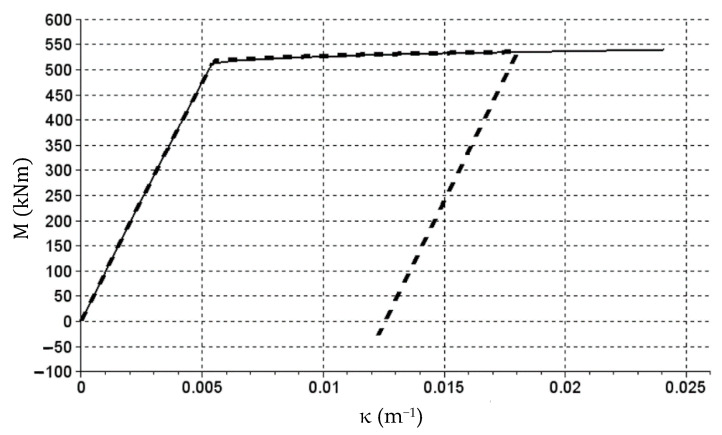
The M(κ) relation graph in cross-sections at beam start and end (thick dashed line) overlapped with the graph from Figure 19.

In the presented example, there were different values of bending moments and curvatures in individual cross-sections. The most significant plastic rotation of cross sections occurred at beam start and end (at terminal supports). The fact that plastic joints were formed only in the most stressed sections is typical for bar structures. The plasticization of the outermost sections is indicated by large value jumps at these locations seen in Figure 23. In Figure 22, the presence of plastic cross sections in these sections is indicated less clearly, but it is due to the limitations of the finite difference method used. Figure 26 shows the M(κ) relation graph in these cross-sections overlapped with the graph from Figure 19.

High compatibility of both graphs can be seen in Figure 26. Some differences occurred at the point of severe changes of graph inclination angle. These differences could be reduced, for example, as a result of reduced load increments.

The analyzed beam was previously solved in study [37]. Calculations performed there took into account stiffness functions determined in accordance with the same algorithm (study [36]), but without using the stiffness matrix description. One load cycle up to q = 50 kN/m was taken into account. At such load, the maximum deflection value of 0.0031073 m was obtained in [37]. Having solved the same problem using the algorithm discussed in this paper, a maximum deflection value of 0.0031061 m was obtained. This means that the relative error of the deflection value is −0.04%. This confirms that the algorithm works correctly as planned.

## 4. Discussion

The purpose of this paper was to present the developed matrix constitutive model of structural materials. The main assumptions of this method and the numerical algorithm to allow for its efficient use were presented in Section 2.

In Section 3, the paper presented calculation examples that confirm the effectiveness of the developed method and show some fields of application. Example B presented two constructed stiffness matrices for the assumed baseline data, with the second matrix (Case 2) referring to the data extracted from the experiment. Despite manual calibration of the stiffness matrix, a good compliance between the tension test simulation results and the baseline data was obtained. The versatility of the method is also apparent here. Stiffness matrices can be constructed for materials with distinct yield strength, for hyper-elastic materials, and material types not considered in this paper.

Examples C and D show simulations of tension, relief, and compression of a sample in axial compressive stress. Obtained simulation results confirm the high versatility of the method as well as the ease of adaptations. In example E, a degradation model was built by introducing a coefficient that depends on strain history, but a similar effect could be obtained by modifying the stiffness matrix accordingly. In Example D, the stiffness matrix was modified to easily account for compressive stresses. The simulation of the tensile processes of the sample resulted in a graph in the form of an exactly closed hysteresis loop, which confirms that the method is correct and accurate.

Examples E and F deal with the analysis of statically indeterminate systems. In these cases, the calculation algorithms shown in Figure 5 and Figure 6 were fully utilized. In Example E, a system of three parallel connected bars that were tensioned with a force that causes plastic deformation was analyzed. After relief, the system did not return to its original position and non-zero strains and stresses remained in the system. Example F was related to a double-span reinforced concrete beam. The stiffness matrices created in this case referred to the complete reinforced concrete beam cross-sections that take into account the concrete matrix and reinforcing bars (in earlier examples the matrices described material stiffness). This is another confirmation of the versatility and adaptability of the developed method. In this case, the finite difference method was used to create the mathematical model of the task, but stiffness matrices can be used along with other numerical methods such as the finite element method. Simulation of the beam behavior under stress causing high plasticization of outermost sections and then during relief was carried out. After the load relief, non-zero displacements and internal forces remained in the beam. The example in question was previously solved for another article. Result comparison confirmed the correct functioning of the stiffness matrix and calculation algorithm. During the simulation, high compatibility of the obtained M(κ) function with the assumed one was obtained (see Figure 26), which is another confirmation of the proper operation of the algorithm.

**Figure 27:**
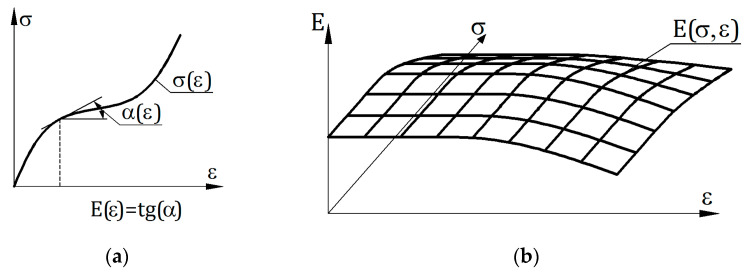
Description of material stiffness: (**a**) classic—stiffness is a function of one variable; (**b**) matrix—stiffness is a function of two variables.

An important advantage of the developed matrix description of material properties is the dependence of the stiffness function on two variables, stress σ and strain ε. The difference between this approach and the classical approach is shown in Figure 27. In the classical approach (Figure 27a), the stiffness function depends on one variable, and it can be represented graphically as a line on a plane graph. Method presented in this paper refers to a description of the stiffness, the graphical interpretation of which is the surface (Figure 27b). This way it is much easier to analyze the loading and unloading processes and take the earlier deformation history of the material into account.

**Figure 28:**
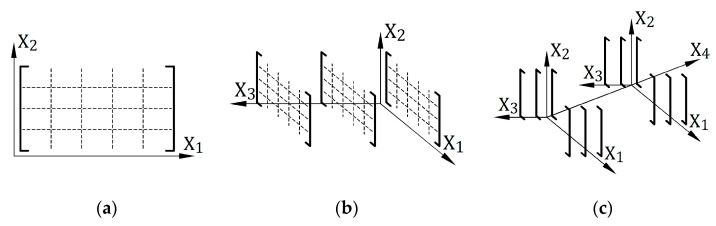
Matrix description of material parameters based on the number of variables: (**a**) two variables—used in the examples in this paper; (**b**) three variables; (**c**) four variables.

The matrix description of materials ensures many more possibilities than those presented in this paper. In particular, it is possible to introduce more variables on which the described parameters will depend. This is explained in Figure 28.

Figure 28a shows a situation where a material parameter depends on two variables. This corresponds to stiffness matrices E(σ, ε) included in this paper. Figure 28b shows the case of a parameter that depends on three variables. This third variable may be, for example, the distortion rate $\dot{\varepsilon }$, which will enable time-dependent analysis. Figure 28c develops the concept with adding another variable. For practical reasons, the cases shown in Figure 28b,c can be prepared by using matrix structures with more than two dimensions. The use of such multidimensional matrices would require appropriate modification of the calculation algorithm discussed in this paper.

The developed method of describing material properties is alternative to classical constitutive models of constitutive materials. It has both advantages (described above) and limitations related to classical models. These limitations include:

- Time-consuming calibration of the stiffness matrix that requires supervision by experienced and dedicated researchers,
- Application limitation to nonlinear issues (using this method in linear problems is possible but inefficient),
- The need to adapt the incremental method parameters of given problem solution to the distribution of characteristic points in vectors X and Y.

Areas of further planned research and activities to improve the method include, among others, the algorithm development for automated calibration of the stiffness matrix. This will enable a quick creation of accurate stiffness matrices based on basic data, e.g., laboratory test results.

Potential areas of application of the method include the analysis of structures made of hyper-elastic materials and structures connected with the ground. The ground behaves in a non-linear and complex way, which is difficult to describe using mathematical expressions, so writing down its properties in the form of a stiffness matrix should give good results. Due to the possibility of direct referencing the stiffness matrix with the results of material laboratory tests, it is possible to apply the method when analyzing structures made of new materials or for which no verified analytical constitutive models exist.

## 5. Conclusions

The concept of matrix description of material properties presented in this paper is an original approach to the creation of nonlinear constitutive models. The distinguishing feature of this concept is the exclusive use of numerical description (in the form of one or more matrices) and taking into account the dependence of the stiffness parameter on at least two variables. The developed method offers the accurate description of the material behavior, at the cost of its workload. The computational examples presented in the paper, incorporating the developed numerical algorithm, confirmed the effectiveness and accuracy of the method. It is primarily developed for the description and analysis of nonlinear materials. In particular, it is suitable for the description of materials where effective analytical constitutive models have not been developed. The proposed approach offers great adaptability for describing different types of materials as well as for the analysis of a variety of structural analysis issues. Stiffness matrices can be used in conjunction with numerical structural analysis methods such as the finite difference method or the finite element method.

## Data Availability

Data sharing is not applicable for this article.
